# Personality in Chimpanzees (*Pan troglodytes*): Exploring the Hierarchical Structure and Associations with the Vasopressin V1A Receptor Gene

**DOI:** 10.1371/journal.pone.0095741

**Published:** 2014-04-21

**Authors:** Robert D. Latzman, William D. Hopkins, Alaine C. Keebaugh, Larry J. Young

**Affiliations:** 1 Department of Psychology, Georgia State University, Atlanta, Georgia, United States of America; 2 Neuroscience Institute, Georgia State University, Atlanta, Georgia, United States of America; 3 Division of Developmental and Cognitive Neuroscience, Yerkes National Primate Research Center, Atlanta, Georgia, United States of America; 4 Center for Translational Social Neuroscience, Division of Behavioral Neuroscience and Psychiatric Disorders, Yerkes National Primate Research Center & Department of Psychiatry and Behavioral Sciences, Emory University School of Medicine, Atlanta, Georgia, United States of America; University of Missouri, United States of America

## Abstract

One of the major contributions of recent personality psychology is the finding that traits are related to each other in an organized hierarchy. To date, however, researchers have yet to investigate this hierarchy in nonhuman primates. Such investigations are critical in confirming the cross-species nature of trait personality helping to illuminate personality as neurobiologically-based and evolutionarily-derived dimensions of primate disposition. Investigations of potential genetic polymorphisms associated with hierarchical models of personality among nonhuman primates represent a critical first step. The current study examined the hierarchical structure of chimpanzee personality as well as sex-specific associations with a polymorphism in the promoter region of the vasopressin V1a receptor gene (*AVPR1A*), a gene associated with dispositional traits, among 174 chimpanzees. Results confirmed a hierarchical structure of personality across species and, despite differences in early rearing experiences, suggest a sexually dimorphic role of *AVPR1A* polymorphisms on hierarchical personality profiles at a higher-order level.

## Introduction

One of the major contributions of recent personality psychology is the finding that traits are related to each other in an organized hierarchy. Indeed, although the five-factor model (FFM; e.g., [1], [2]) is the most widely-used taxonomy for organizing personality traits among human and nonhuman primate populations, well-replicated findings have led to an increased understanding of how two-, three-, four-, and five-factor models of personality are hierarchically related among both human adults (e.g., [3], [4]) and youth (e.g., [5], [6]). Although there has been some interest in the idea of a general factor of personality among nonhuman primates (e.g., [7]), researchers have yet to explicitly investigate the hierarchical nature of personality among nonhuman primates. Such investigations are critical for confirming the cross-species nature of trait personality and in further illuminating personality as neurobiologically-based and evolutionarily-derived dimensions of primate disposition. Investigations of potential genetic polymorphisms associated with personality among nonhuman primates represents a critical first step in potentially addressing some of these concerns. The current study aimed to address this gap in the literature by examining the hierarchical structure of personality in a chimpanzee sample. In addition we examined potential genetic underpinnings of personality at various levels of the hierarchy. Specifically, given previous findings of associations between personality and variation in the vasopressin V1a receptor gene (*AVPR1A*) in both humans (e.g., [8]) and chimpanzees (e.g., [9]), we chose to examine associations with this gene.

### Chimpanzee Personality

Converging empirical literatures have found largely similar core personality traits in nonhuman animals as those found among humans (e.g., FFM; for a review, see [1]). For example, among chimpanzees, humans’ closest primate relative, a similar five factor model of personality has been found, with the addition of a sixth, Dominance dimension (see [10]). Factor-analytic results are not unequivocal, however. Although results of factor-analytic studies have found the FFM plus Dominance emerging [11], more recent work in a larger sample of chimpanzees from different settings [12] has failed to extract these six factors and has instead found four: Dominance, Extraversion, Conscientiousness, and Agreeableness; Neuroticism and Openness/Intellect did not emerge. Regardless of the ultimate number of factors that emerge, the existence of largely parallel dispositional traits among both human and nonhuman primates is clear. In addition to research confirming the existence of largely parallel traits in human and nonhuman primates, recent work has begun to investigate whether a general factor of personality is present across a number of nonhuman primate species [7]. Nonetheless, research to date has yet to explicitly investigate the existence of a parallel hierarchical structure of personality among nonhuman animals as has been found in humans.

### Hierarchical Nature of Personality

Research indicates that innate individual differences, often referred to as temperament, form the basis for personality traits organized into robust, higher order personality dimensions, or “metatraits” [4]. Consistent with this understanding, Clark (2005) asserted that adult personality traits emerge through differentiation from three (“Big Three”) largely innate biobehavioral temperament dimensions [13]. Two of these dimensions are affective, namely, Negative and Positive Emotionality, and the third dimension, Disinhibition (vs. Constraint), is a regulatory system that plays a role in the perception and interpretation of incoming stimuli [14] [15]. Recent structural work revealed robust hierarchical associations between these three temperament traits and dimensions of the FFM (for review, see [16]).

The FFM dimensions can be understood as lower order components of the Big Three [4]. Specifically, Neuroticism, along with some components of low Agreeableness, combine to form Negative Emotionality at the higher order level; Extraversion and Openness combine to form Positive Emotionality; and low Agreeableness and low Conscientiousness combine to form Disinhibition. Further, going the other direction (i.e., above the Big Three), it has been shown that both the FFM and the Big Three have two consistent higher-order factors or “metatraits.” Specifically, low Disinhibition via FFM Agreeableness and Conscientiousness as well as low Negative Emotionality or FFM Neuroticism is captured by Alpha/Stability whereas Positive Emotionality or FFM Extraversion is captured by Beta/Plasticity [17], [4]. Among human youth, results of both exploratory and confirmatory factor analyses has confirmed the existence of this hierarchical structure cross-nationally [6].

Although well-established among humans, this hierarchical structure of personality has not yet been examined among nonhuman animals. Given the growing interest in the study of trait personality within nonhuman animals (e.g., [18]), and the elucidation of a similar trait structure among different species (e.g., [1], [10], [19], [12]), such an investigation is a logical next step. As was argued more than 80 years ago, research on personality among nonhuman animals is critically important in elucidating common biological systems underlying personality [20]. Despite the recent interest in comparative examinations of personality among nonhuman primates, however, the vast majority of studies to date have focused on the description and construction of personality and personality assessment instruments. Although some work has examined the heritability of personality traits (e.g., [21]) as well as neuroanatomical correlates (e.g., Latzman, Hecht, Freeman, Schapiro, & Hopkins, manuscript under review) among nonhuman primates, there is a surprising scarcity of research on potential specific genetic factors associated with these individual differences (for exceptions, see [9], [22]). As such, our understanding of the mechanisms underlying the expression of personality dimensions in chimpanzees is largely unknown. Among both humans and great apes, however, there is evidence dimensions of personality are heritable [23], [24] underscoring the importance of considering potential genetic contributors to individual variation in personality.

### Vasopressin V1a Receptor Gene (*AVPR1A*)

Vasopressin is a neuropeptide with multiple physiological functions that has been strongly implicated in the development and evolution of complex social behavior in mammals [25]. Across numerous species, *AVPR1A* has been found to be expressed in the brain and associated with several social behaviors including aggression, territoriality, and pair bonding behaviors among voles, particularly among males (e.g., [26], [27]). The behavioral effects of vasopressin have been found to be mediated predominantly by the V1a subtype of the vasopressin receptor, although it should be noted that the V1b subtype has received much less attention in the literature. Both species differences and individual variation in the distribution of *AVPR1A* in the brain are thought to underlie inter- and intraspecies variation in social behavior [27], [28], behaviors known to be particularly relevant to individual variation in trait dispositions.

With regard to primates, although less is known, recent studies with humans suggest associations with a similar repetitive element in the *AVPR1A* promoter and social behavior, including at first intercourse [29], altruism [30], and pair bond relationships [31]. Further, *AVPR1A* promoter polymorphisms have been found to be associated with increased Novelty Seeking, decreased Harm Avoidance [31], and increased Reward Dependence [8]. Harm Avoidance is strongly correlated with FFM Neuroticism and Novelty Seeking and Reward Dependence are strongly correlated with FFM Extraversion [32]. Among chimpanzees, Hopkins et al. (2012) found that Dominance and Conscientiousness scales were associated with polymorphic variation in *AVPR1A*, particularly among males. Specifically, for those chimpanzees with one copy of the long allele DupB^+/−^ (versus those homozygous for the short allele, DupB^−/−^), males had higher Dominance and lower Conscientiousness scores than females [9].

Many of the association studies mentioned above focused on a polymorphic repetitive element known as RS3. In humans, the RS3 repeat region is housed within a larger, ∼350 bp tandem duplicated region. The first of these duplicated regions, DupA, spans −3730 to −4074 bp relative to the transcription start site and contains a GT_20–26_ microsatellite, known as STR1. The second block, DupB, spans −3382 to −3729 bp and contains the complex microsatellite, RS3 ((CT)_6–14_(GT)_8–24_). Approximately 65% of the AVPR1A alleles in chimpanzees have a complete deletion of the DupB region, leading to a 357 bp difference between the DupB^+^ and Dup^−^ alleles. The deletion of RS3 in some individuals makes this species ideal for assessing the potential role of the *AVPR1A* gene, and more specifically RS3, on aspects of sociality such as personality.

In addition to the unique *AVPR1A* polymorphism, the chimpanzees in the current study were raised in different environments early in life. To what extent these different rearing experiences might influence the development of personality in chimpanzees is a critically important question, but has yet to be empirically considered. Indeed, a large literature documents the direct effects of early social experiences on development in humans and many animals, including primates [33]. Further, although not previously studied with regard to the *AVPR1A* polymorphism, early rearing experiences have been found to interact with other candidate genes in the prediction of behavioral development in both human and nonhuman primates [34], [35]. Lastly, potentially due in part to a higher expression of arginine vasopressin in males [36], the vasopressin systems in the brain have been found to be sexually dimorphic and thought to regulate social behaviors in sex-specific ways [37]. Given these previous sexually dimorphic findings for the vasopressin system broadly, as well as recent findings of sexually-dimorphic associations in the *AVPR1A* with personality (e.g., [9]), it is important for sex-specific associations to be examined.

### Current Study

The current study had two primary aims. First, we examined the hierarchical nature of personality in a sample of chimpanzees. Second, given previous research suggesting the importance of considering early rearing environment in genetic studies in monkeys (e.g., [38]), we simultaneously examined the effects of *AVPR1A* and early rearing experiences on chimpanzee personality in service of isolating the role of experience from genetic factors. Specifically, we assessed whether differences in personality at the various levels of the hierarchy were influenced by a) the subjects rearing experience and/or b) the presence or absence of the RS3-containing DupB element in the *AVPR1A* 5’ flanking region. Given converging evidence of a hierarchical personality structure in humans (e.g., [16]), as well as clear parallels between chimpanzee and human personality (e.g., [11], [12]), we expected a similar hierarchical structure to emerge with regard to chimpanzee personality. Given previous findings of sexually-dimorphic associations in the *AVPR1A* (e.g., [9]), along with previous findings suggesting a higher expression of arginine vasopressin in males (e.g., [36]), expected to find sexually-dimorphic variation in the *AVPR1A* in chimpanzees linked to the hierarchical levels in personality structure, particularly among those that retained the items linked to Dominance and Conscientiousness as was previously found in chimpanzees (i.e., [9]). We expected sexually-dimorphic associations with these dimensions specifically given associations between these dimensions and aggression and territoriality, behaviors that occur in the context of social interactions previously found to be associated with vasopressin in sex-specific ways (e.g., [25], [26]). Moreover, based on previous reports on the effect of rearing on personality rating in chimpanzees [39], we hypothesized that these effects would be evident independent of the chimpanzees’ early social rearing experience.

## Method

### Subjects

Chimpanzees were members of the colony of apes housed at the Yerkes National Primate Research Center. Personality ratings were available in 174 adult and sub-adult chimpanzees including 108 females and 66 males. Within the 174 subjects, DNA samples were available in 116 chimpanzees including 72 subjects (62.07%) with the DupB^−/−^ and 44 (37.93%) DupB^+/−^ genotypes. Seventy-nine chimpanzees were in the original study by Hopkins et al. (2012); thus, this study included genotype data from 37 new chimpanzee subjects. Animals experienced a variety of early rearing experiences with 46 being mother-reared, 50 human nursery-reared, and 20 wild-born. Nursery-reared chimpanzee were separated from their mothers within the first 30 days of life, due to unresponsive care, injury, or illness [40], [41] and placed in incubators, fed standard human infant formula, and cared for by humans until they could sufficiently care for themselves. At this time, they were placed with other infants of the same age until they were three years of age [40], [41]. At three years of age, the nursery-reared chimpanzees were integrated into larger social groups of adult and sub-adult chimpanzees. Importantly, chimpanzees were nursery-reared because their biological mothers did not exhibit adequate maternal care at birth requiring intervention to protect the infants’ well-being. Thus, the chimpanzees in this study were not nursery-reared with the goal of subsequently determining the effects of early life experiences on personality. These data are therefore opportunistic and retrospective and we took advantage of this to determine whether early rearing experiences might have consequences on personality. Mother-reared chimpanzees were raised by their mother for at least 2.5 years of life and were raised in ‘nuclear’ family groups of chimpanzees, with group sizes ranging from 4 to 20 individuals. Finally, one cohort of chimpanzees were wild-caught and subsequently brought to a captive environment. In 1973, there was a ban on the importation of chimpanzees from the wild and therefore these apes were relatively older individuals.

### Ethics Statement

All aspects of the research complied with the American Psychological Association’s Guidelines for Ethical Conduct in the Care and Use of Nonhuman Animals in Research [42], followed the Institute of Medicine guidelines for research with chimpanzees, and was done with the approval of the Georgia State University Institutional Care and Use Committee (A3914-01). All the chimpanzees were housed in social groups ranging from two to 12 individuals. The chimpanzees are housed in indoor-outdoor compounds and have access to both enclosures 24 hours a day. During the winter seasons, the indoor facilities are heated while air conditioning is provided in the hotter summer months. Lighting in the outdoor facility follows the typical seasonal cyclic change in sunrise and sunset. Standard tungsten lighting is provided in the indoor facility and the lights are on a 12 hour on-off cycle. The chimpanzees are fed twice daily with a diet that consists of fruits, vegetables and commercially produced primate chow. Environmental enrichment, such as simulated tool use tasks or other non-nutritive substrates, were provided to the chimpanzees on a daily basis. At no time were the subjects ever food or water deprived. We thank the animal care and enrichment staff for maintaining the health and wellbeing of the apes. The supporting ARRIVE checklist is available as Checklist S1.

### Personality Assessment

The Chimpanzee Personality Questionnaire (CPQ; [11]) is a personality instrument consisting of 43 adjectives followed by one to three sentences that define the adjective consistent with dictionary definitions and within the context of chimpanzee behavior. Ratings are made individually on a seven-point Likert-type scale (1 =  “Displays either total absence or negligible amounts of the trait; 7 =  “Displays extremely large amounts of the trait”) and raters are instructed to base their ratings on overall impressions and not estimated frequencies of particular behaviors. While previous factor-analytic studies have found a six-factor solution that includes the five FFM factors (i.e., Extraversion, Conscientiousness, Agreeableness, Neuroticism, and Openness) plus Dominance [11], [43], others have failed to extract the sixth factor and have instead found five [12]. Reliability of the CPQ has been found to be adequate both in terms of inter-rater reliability as well as internal consistency [11], [12]. Consistent with previous research using the CPQ, mean ratings across raters were used for all analyses. As has been previously reported, mean interrater reliability across all items was.26 with range of.06 to.44, respectively [12].

### DNA Extraction, Genotyping and Analysis

DNA samples were isolated from buccal swabs or blood samples using Puregene DNA purification system (Gentra, Minneapolis, MN, USA) as described by Donaldson et al. (2008). Following extraction, stock DNA was separated into three aliquots: one for onsite storage at −80°C, one for offsite storage, and a working stock for genotyping. Samples were tracked via a secure Filemaker Pro 8 database that linked sample codes for each aliquot, demographics for each subject, DNA quantification and purity analysis results, and genotype data.

Each individual was genotyped for the *AVPR1A* DupA/B region using the primers and conditions reported in previous studies with slight modifications [44]. Briefly, we used forward primer 5′-GCATGGTAGCCTCTCTTTAAT and a reverse primer of 5′- CATACACATGGAAAGCACCTAA with an annealing temperature of 57°C for 30 cycles: 95°C, 5 min; 30×(95°C, 30 seconds; 57°C, 30 seconds; 72°C, 3 min; 72°C, 10 min; 4°C, hold). Polymerase chain reaction (PCR) amplification was undertaken using the Epicentre Failsafe kit using premixH (Illumina Inc., Madison, WI, USA) according to the manufacturer’s directions. Genotyping was performed in a volume of 20 µl containing 20 ng target genomic DNA. PCR products were resolved on a 2% agarose gel (SeaKem Agarose LE, Lonza, Basel, Switzerland) at 100 V for 45 min with a 100-bp DNA ladder (New England Biolabs, Ipswich, MA, USA) in tris-borate-EDTA (TBE). The DupB-containing allele resulted in a band of ∼900 bp, while the DupB minus allele was ∼570 bp long, and genotypes were visually assigned [44]. All genotypes were run in duplicate with gel analysis and were checked before the data set was finalized.

### Data Analysis

Data analysis was performed in a two-step procedure. First, to address the hierarchical nature of personality among chimpanzees, CPQ items were subjected to a series of principal components analyses for investigating the hierarchical structure of personality (see [45]). Specifically, a series of orthogonally-rotated (varimax) principle components analyses were performed in an iterative manner extracting first two principal components (given the previously elucidated hierarchical structure among humans; [16]) and saving the regression-based factor scores, followed by three principle components and so on. Next, to examine how lower levels of the hierarchy emerged from higher levels, we then correlated the saved factor scores between levels of the personality hierarchy. Based on this approach, a hierarchical structure of personality was constructed by using the correlations as path estimates between each subsequent level of hierarchy and the proceeding level. For example, path estimates were examined between each of the two factors at the two-factor level and the three factors at the subsequent three-factor level, and so on. This approach has been successfully utilized in the past for similar investigations in humans (e.g, [5], [6]). To determine the number of factors to extract, we derived eigenvalue Monte Carlo p values (e.g., parallel analysis; [46]) as parallel analysis has been shown to perform well in identifying the number of factors in an exploratory factor analysis (EFA) model [47], [48]. These analyses suggested that a four-factor solution best fit the data. Nonetheless, as five-factors are consistently found in the human literature and up to six factors have emerged among chimpanzees (e.g., [11], [43]), albeit with smaller sample sizes in general, we decided to be more inclusive and extracted five factors.

Next, to examine associations between personality at various levels of the hierarchy and *AVPR1A* polymorphisms, we used multivariate analysis of variance to examine associations between the *AVPR1A* DupB genotype and personality at various levels of the personality hierarchy. Specifically, we used factor scores from the hierarchical analyses as the dependent measures and sex, rearing history and genotype as the between group variables. Analysis of Variance (ANOVA) was then used to examine potential two-way sex by genotype interactions. Prior to the inferential statistical analyses, we calculated relatedness coefficients for each subject to all individuals in the known pedigree. The relatedness coefficients were used as covariates in all analyses to adjust for genetic relatedness among individuals within the sample. Consistent with existing models biologically-based of personality, and given previous research with a subset of animals included in the current study has already examined *AVPR1A* and personality at the four- and five-factor levels, albeit at the scale-level (see [9]), we chose to examine associations only at the two- and three-factor levels.

## Results

### Hierarchical Structure of Chimpanzee Personality

**Figure 1:**
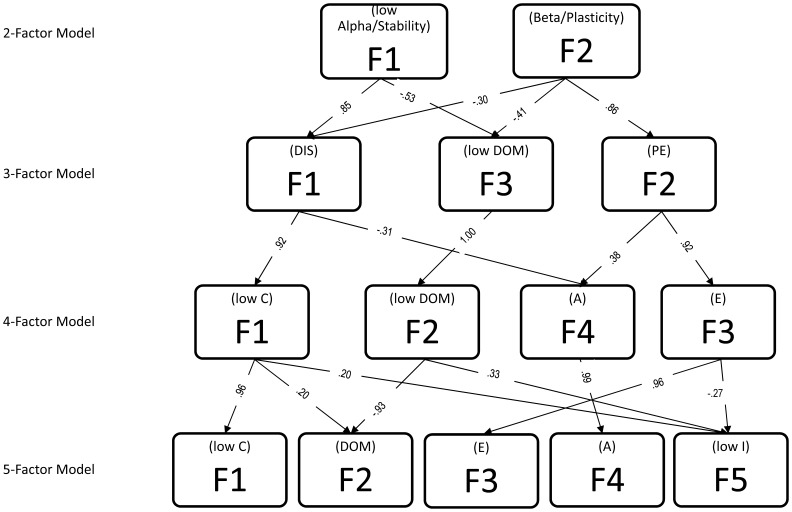
Hierarchical structure of chimpanzee personality. *Note.* Order of factors extracted indicated by factor labels (e.g., F1 = first factor extracted). Dis = Disinhibition. NE/Dom = Negative Emotionality/Dominance. PE = Positive Emotionality. C = Conscientiousness. Dom = Dominance. A = Agreeableness. E = Extraversion. I = Intellect. Only significant (p<.05) paths shown.

Results of item-level factor analyses of the 43 CPQ items provide support for correspondence of the hierarchical structure of personality established in humans (e.g., [3], [5], [6], [16]). Specifically, as shown in Figure 1, the two-factor level appears to describe dimensions that resemble (low) Alpha/Stability and Beta. Specifically, as shown in Table 1, the (low) Alpha/Stability factor is anchored by items including: Aggression, Bullying, Reckless, Gentle(−), Defiant; the Beta/Plasticity factor is anchored by items including: Depressed(−), Sociable, Solitary(−), Inquisitive, Playful, and Affectionate. At the three-factor level (see Table 2), the three dimensions that emerge are similar to the “Big Three” of Disinhibition (vs. Constraint), Positive Emotionality, and Negative Emotionality with Alpha/Stability breaking into Disinhibition and (low) Dominance. It should be noted, however, that chimpanzee Negative Emotionality appears to be a combination of traditional human Negative Emotionality items (e.g., Fearful, Cautious) and Dominance (e.g., dependent(−) and submissive(−)) items. As the strongest loading items are the overtly dominant items, we chose to label this factor Dominance. The Disinhibtion factor is anchored by items including: Impulsive, Erratic, Reckless, Excitable, and Irritable; the Positive Emotionality factor is anchored by items including: Playful, Inquisitive, Solitary(−), Lazy(−), and Sociable; and the low Dominance factor is anchored by items including: Dependent, Submissive, Timid, Independent(−), and Fearful. At the next level, as shown in Table 3, a fourth factor resembling Agreeableness breaks off with contributions from three-factor Disinhibition(−) and Positive Emotionality. Specifically, the Agreeableness factor is anchored by items including: Protective, Helpful, Sympathetic, Sensitive, and Affectionate. Lastly, at the five-factor level, a factor similar to low Intellect/Openness emerged with contributions from low Conscientiousness, low Dominance, and Extraversion(−). The low Intellect/Openness factor is anchored by items including: Disorganized, Intelligent(−), and Clumsy (see Table 4).

**Table 1:** Varimax Rotated exploratory factor analysis of Chimpanzee Personality Questionnaire: Two factor solution.

	Factor	
Item	1 (low “Alpha”/“Stability”)	2 (“Beta”/“Plasticity”)
Aggression	**.86**	−.01
Bullying	**.84**	.08
Reckless	**.81**	.03
Gentle	**−.78**	.19
Defiant	**.74**	.02
Dominant	**.72**	.22
Stingy	**.71**	−.06
Cautious	**−.70**	−.14
Irritable	**.70**	−.42
Impulsive	**.67**	−.10
Jealous	**.65**	.10
Submissive	**−.64**	−.37
Sympathetic	**−.64**	.34
Persistent	**.63**	.31
Erratic	**.58**	−.48
Excitable	**.57**	−.17
Dependent	**−.54**	−.26
Stable	**−.51**	.29
Independent	**.50**	.22
Sensitive	−.49	.41
Manipulative	.48	.18
Decisive	.42	.33
Predictable	−.34	.22
Fearful	−.34	−.28
Imitative	−.15	.08
Depressed	−.03	**−.79**
Sociable	−.24	**.75**
Solitary	−.01	**−.72**
Inquisitive	.09	**.71**
Playful	.07	**.67**
Affectionate	−.47	**.66**
Disorganized	.11	**−.65**
Timid	−.45	**−.63**
Intelligent	.07	**.61**
Clumsy	−.02	**−.57**
Lazy	−.24	**−.55**
Friendly	**−.54**	**.54**
Inventive	.20	**.50**
Active	.32	.46
Autistic	.12	−.41
Unemotional	−.39	−.39
Helpful	−.35	.37
Protective	−.19	.32

*Note. N = *174. Loadings ≥.50 shown in **boldface.** The order in which factors were extracted is reflected in the factor numbers.

**Table 2:** Varimax Rotated exploratory factor analysis of Chimpanzee Personality Questionnaire: Three factor solution.

	Factor		
Item	1 (DIS)	2 (PE)	3 (NE/low DOM)
Impulsive	**.77**	.14	−.03
Erratic	**.77**	−.24	.12
Reckless	**.76**	.15	−.31
Excitable	**.76**	.14	.14
Irritable	**.76**	−.29	−.13
Aggressive	**.75**	.04	−.42
Stable	**−.74**	−.03	−.23
Gentle	**−.72**	.14	.34
Bullying	**.66**	.06	**−.53**
Defiant	**.65**	.08	−.34
Sympathetic	**−.63**	.28	.21
Jealous	**.60**	.20	−.26
Stingy	**.58**	−.07	−.42
Friendly	**−.56**	**.52**	.16
Predictable	**−.51**	−.01	−.17
Sensitive	−.48	.40	.19
Persistent	.47	.32	−.41
Helpful	−.37	.34	.10
Manipulative	.35	.17	−.34
Autistic	.34	−.22	.30
Playful	.08	**.84**	.06
Inquisitive	.02	**.80**	−.08
Solitary	.07	**−.78**	.08
Lazy	−.27	**−.75**	−.02
Sociable	−.35	**.72**	−.06
Depressed	.19	**−.71**	.30
Active	.40	**.71**	.08
Affectionate	**−.54**	**.61**	.06
Inventive	.09	**.51**	−.21
Unemotional	−.33	−.48	.18
Intelligent	−.18	.45	−.39
Protective	−.25	.27	−.02
Dependent	−.12	.04	**.81**
Submissive	−.25	−.13	**.81**
Timid	−.05	−.39	**.74**
Independent	.12	−.06	**−.74**
Fearful	.04	.03	**.70**
Dominant	.41	.06	**−.70**
Decisive	.08	.11	**−.65**
Cautious	−.46	−.04	**.58**
Disorganized	.42	−.40	.42
Imitative	.07	.32	.42
Clumsy	.26	−.37	.42

*Note. N = *174. DIS = Disinhibition. PE = Positive Emotionality. NE/low Dom = Negative Emotionality/low Dominance. Loadings ≥.50 shown in **boldface.** The order in which factors were extracted is reflected in the factor numbers.

**Table 3:** Varimax Rotated exploratory factor analysis of Chimpanzee Personality Questionnaire: Four factor solution.

	Factor			
Item	1 (low C)	2 (low DOM)	3 (E)	4 (A)
Excitable	**.78**	.15	.10	−.07
Impulsive	**.77**	−.01	.14	−.15
Erratic	**.76**	.13	−.24	−.22
Irritable	**.74**	−.12	−.27	−.26
Aggressive	**.74**	−.40	.08	−.26
Defiant	**.71**	−.33	.04	−.02
Reckless	**.70**	−.28	.22	−.28
Bullying	**.64**	**−.52**	.09	−.19
Jealous	**.63**	−.25	.18	−.03
Stingy	**.61**	−.41	−.08	−.10
Stable	**−.61**	−.26	−.15	.43
Gentle	**−.57**	.32	−.02	**.53**
Persistent	**.54**	−.40	.27	.09
Manipulative	**.52**	−.34	.01	.32
Autistic	.44	.29	−.33	.12
Predictable	−.40	−.19	−.11	.35
Dependent	−.11	**.81**	.00	.11
Submissive	−.26	**.80**	−.15	.05
Independent	.17	**−.74**	−.08	.02
Timid	−.02	**.72**	−.45	.05
Fearful	.07	**.70**	−.03	.09
Dominant	.49	**−.69**	.01	.06
Decisive	.17	**−.65**	.05	.15
Cautious	−.34	**.56**	−.17	.37
Disorganized	.38	.43	−.39	−.22
Imitative	.20	.42	.19	.34
Playful	.08	.08	**.85**	.14
Lazy	−.19	−.05	**−.82**	.07
Solitary	.09	.06	**−.80**	−.12
Active	.33	.11	**.78**	−.11
Inquisitive	.07	−.06	**.75**	.25
Depressed	.21	.28	**−.75**	−.11
Sociable	−.21	−.06	**.59**	**.52**
Unemotional	−.30	.16	**−.51**	.04
Clumsy	.34	.41	−.47	.07
Inventive	.19	−.20	.42	.30
Protective	.06	−.05	−.03	**.80**
Helpful	−.10	.07	.09	**.73**
Sympathetic	−.41	.19	.06	**.69**
Sensitive	−.30	.17	.23	**.58**
Affectionate	−.39	.05	.46	**.57**
Friendly	−.42	.15	.37	**.54**
Intelligent	−.03	−.40	.33	.43

*Note. N = *174. low C = low Conscientiousness. low Dom = low Dominance. E = Extraversion. A = Agreeableness. Loadings ≥.50 shown in **boldface.** The order in which factors were extracted is reflected in the factor numbers.

**Table 4:** Varimax Rotated exploratory factor analysis of Chimpanzee Personality Questionnaire: Five factor solution.

	Factor				
Item	1 (low C)	2 (Dom)	3 (E)	4 (A)	5 (low I)
Excitable	**.84**	−.06	.07	−.01	−.03
Impulsive	**.77**	.13	.13	−.09	.05
Irritable	**.75**	.21	−.30	−.19	.05
Erratic	**.74**	.06	−.19	−.13	.33
Stable	**−.71**	.19	−.10	.38	.00
Aggressive	**.65**	**.54**	.08	−.16	.08
Defiant	**.65**	.44	.03	.03	−.01
Reckless	**.65**	.42	.23	−.23	.08
Jealous	**.58**	.35	.17	.01	.01
Gentle	**−.55**	−.40	.01	.49	−.03
Stingy	**.54**	**.50**	−.10	−.05	−.01
Predictable	−.47	.15	−.08	.32	−.02
Autistic	.47	−.22	−.32	.18	.13
Submissive	−.12	**−.84**	−.14	.05	.12
Dominant	.33	**.81**	.04	.09	.05
Dependent	−.02	**−.78**	.05	.12	.22
Independent	.06	**.73**	−.11	.01	−.17
Fearful	.21	**−.72**	−.05	.10	.00
Timid	.04	**−.66**	−.38	.10	.38
Cautious	−.26	**−.63**	−.17	.35	.01
Bullying	**.55**	**.63**	.09	−.14	.01
Decisive	.15	**.55**	−.07	.12	−.47
Persistent	.46	.48	.26	.12	−.08
Manipulative	.41	.46	.05	.36	.07
Playful	.05	−.02	**.88**	.12	−.01
Active	.31	.00	**.82**	−.09	.08
Solitary	.11	−.07	**−.81**	−.09	.12
Lazy	−.22	.02	**−.81**	.09	.13
Depressed	.23	−.22	**−.72**	−.05	.28
Inquisitive	.08	.04	**.72**	.22	−.27
Sociable	−.28	.06	**.62**	.49	−.08
Inventive	.17	.20	.38	.29	−.22
Protective	−.04	.09	.02	**.80**	.04
Helpful	−.12	−.12	.08	**.71**	−.16
Sympathetic	−.43	−.25	.08	**.65**	−.06
Sensitive	−.22	−.34	.14	**.52**	−.43
Affectionate	−.40	−.12	.46	**.51**	−.19
Friendly	−.45	−.18	.42	**.50**	−.03
Imitative	.19	−.33	.25	.37	.19
Disorganized	.31	−.18	−.23	−.12	**.72**
Intelligent	.02	.21	.17	.36	**−.69**
Clumsy	.25	−.18	−.31	.16	**.68**
Unemotional	−.39	−.07	−.38	.07	.49

*Note. N = *174. low C = low Conscientiousness. Dom = Dominance. E = Extraversion. A = Agreeableness. low I = low Intellect. Loadings ≥.50 shown in **boldface.** The order in which factors were extracted is reflected in the factor numbers.

### *AVPR1A,* Rearing and Personality

#### Two-Factors

**Figure 2:**
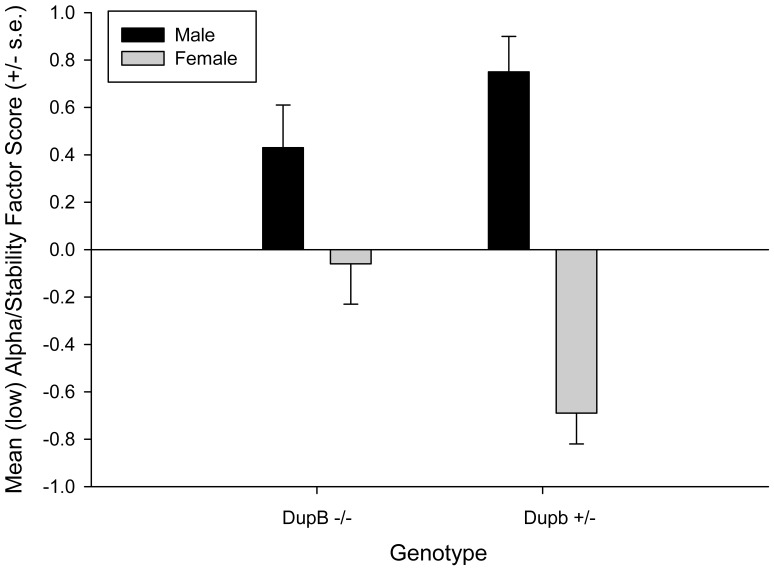
Sex by AVPR1A interactions on (low) Alpha/Stability. *Note.* Mean factor scores (± SE) for the (low) Alpha/Stability factor score for male and female DupB −/− and DupB +/− chimpanzees.

Results of the MANOVA revealed a significant two-way interaction between sex and genotype *F* (2, 95) = 4.75, *p*<.02. Subsequent univariate F-tests revealed a significant two-way interaction between sex, rearing history, and genotype for the (low) Alpha/Stability factor F (1, 96) = 9.54, p<.005. The mean (low) Alpha/Stability factor scores in DupB ^−/−^ and DupB^+/−^ males and females are shown in Figure 2. Tukey’s post-hoc analysis indicated that DupB ^−/−^ females evidenced average levels and Dup^−/−^ males evidenced relatively higher levels. In contrast, DupB^+/−^ males had significantly higher (low) Alpha/Stability scores than DupB^+/−^ females who were reported to have almost 1 SD lower low (Alpha/Stability scores) than average. No significant main effects or interactions were found for the Beta/Plasticity factor.

#### Three-Factors

**Figure 3:**
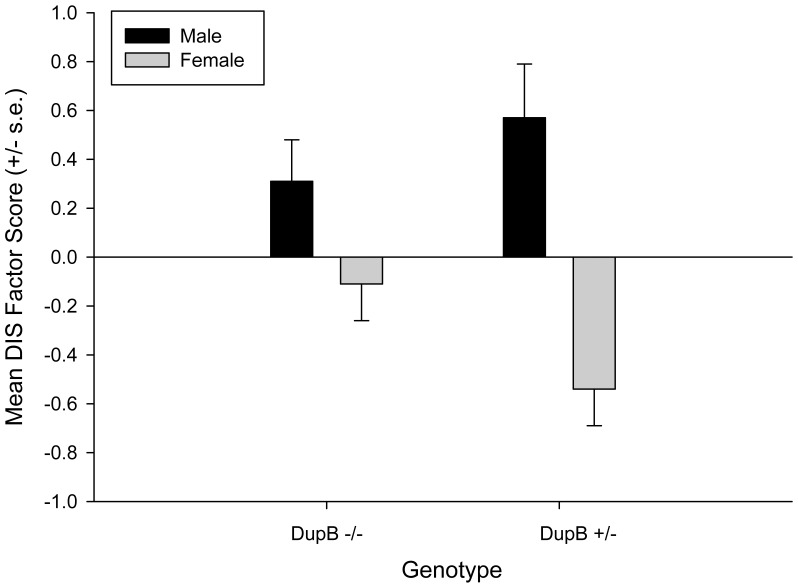
Sex by AVPR1A interactions on Disinhibition. *Note.* DIS = Disinhibition. Mean factor scores (± SE) for the Disinhibition factor score for male and female DupB −/− and DupB +/− chimpanzees.

**Figure 4:**
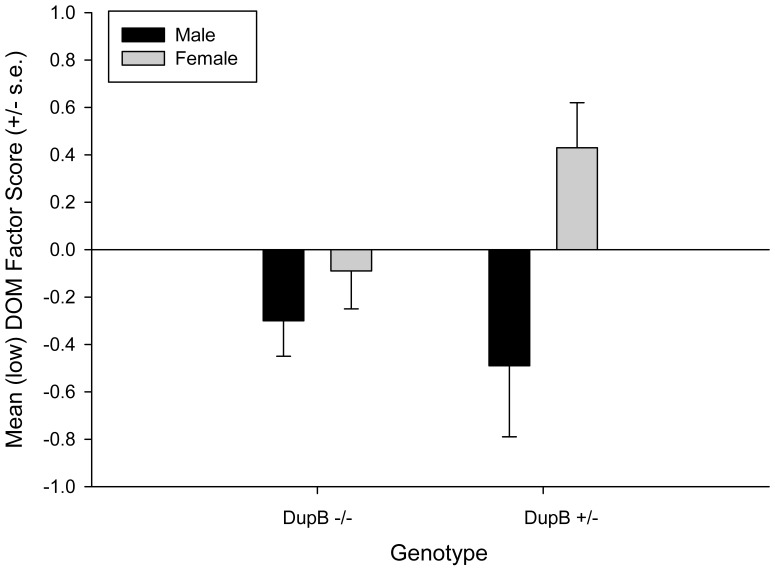
Sex by AVPR1A interactions on (low) Dominance. *Note.* DOM = Dominance. Mean factor scores (± SE) for the (low) Dominance factor score for male and female DupB −/− and DupB +/− chimpanzees.

For this analysis, we repeated the MANOVA but used the three factor structure scores as the dependent measures while sex, rearing history, and genotype again served as the between group factors. As with the previous analysis, there was a significant two-way interaction between sex and genotype *F* (3, 94) = 4.47, *p*<.03. Univariate F-tests revealed significant two-way interactions for the Disinhibition *F* (1, 96) = 4.64 *p*<.04 and Dominance *F* (1, 96) = 4.52, *p*<.04 factors. The mean factor scores for male and females DupB^−/−^ and DupB^+/−^ are shown in Figures 3 and 4. Post-hoc analysis indicated no significant differences between DupB^−/−^ males and females for either factor; however, for Dup^+/−^ individuals, males had significantly higher Disinhibition and Dominance scores than females.

## Discussion

The current study represents the first investigation to date of the hierarchical structure of chimpanzee personality and potential genetic associations. Results confirm a similar hierarchical structure of higher-order personality traits among chimpanzees as has been found among humans (e.g., [3], [5], [6], [16]). Further, we found *AVPR1A* to be associated with the Alpha/Stability metatrait as well as related to lower-order traits including Disinhibition and Dominance. This association was not found for Beta/Plasticity or related traits, suggesting a unique influence of this polymorphism on Alpha/Stability and related traits. Further, consistent with expectations, as described in more detail below, associations were found to vary by sex. Rearing experiences of the chimpanzees did not have a significant effect on personality nor did it interact with the other independent variables, a finding consistent with at least one other report in chimpanzees [39].

### Hierarchical Nature of Chimpanzee Personality

Analagous to recent findings of a hierarchical nature of personality among both human adults (e.g., [3], [4]) and youth (e.g., [5], [6]), the current study reveals a largely parallel structure in chimpanzees. Alpha/Stability and Beta/Plasticity emerged at the highest level of the hierarchy with Disinhibition and Dominance most strongly emerging from Alpha/Stability and Positive Emotionality most strongly emerging from Beta/Plasticity. Additionally, similar to Markon et al (2005), Beta/Plasticity was also characterized by nonneglible negative loadings of Negative Emotionality, in addition to Disinhibition(−). At the next lower level, Disinhibition then clearly becomes low Conscientiousness and, more weakly, low Agreeableness, Dominance remains Dominance, and Positive Emotionality breaks into Extraversion most strongly, but also Agreeableness. At the five-factor level, all four traits emerged largely consistent with the four-factor level but low Intellect emerged with relatively small contributions from Conscientiousness(−), Dominance, and Extraversion(−). It is important to note that the distinctiveness of Intellect in the current study as well as the failure to extract a Neuroticism factor may be a result of the small number of potential loadings on these factors (see [49] for a discussion). Regardless, taken together, these findings confirm the presence of a largely conserved hierarchical structure of personality across species, verifying the evolutionary and neurobiological basis of these general dispositional traits and the ways in which they are associated with each other across various trait models.

### Chimpanzee Personality and *AVPR1A*

The current study suggests a hereditary nature of personality traits in chimpanzees. As expected, vasopressin was differentially associated with various chimpanzee personality traits at different levels of the personality hierarchy. Specifically, *AVPR1A* was associated with Alpha/Stability, but not Beta/Plasticity, at the two-factor level of the hierarchy and, consistent with the hierarchical nature of these traits, with both Dominance and Disinhibition at the three-factor level. And, as with previous findings with a subset of the current sample using scale-level scores (i.e., [9]) these associations were found to be sexually dimorphic. Specifically, while homozygous DupB^−/−^ males and females did not differ, males and females diverged if they possessed a DupB^+^ allele with males evidencing lower levels of Alpha/Stability at the two-factor level and higher levels of Disihibition at the three-factor level. Interestingly, however, the heterozygous genotype was associated with lower level of Dominance for males and higher levels of Dominance for females. That is, at the three-factor level, the form of the genotype by sex interactions are the opposite of each other for Disihibition and Negative Emotionality. This is consistent with previous findings with regard to Dominance and Conscientiousness [9], traits that emerge from Dominance and Disinhibition at a lower level of the hierarchy.

Male-female differences are not surprising as vasopressin systems in the brain have been found to be sexually dimorphic and thought to regulate social behaviors in sex-specific ways [37]. One potential explanation for these findings may be the importance of vasopressin in amygdala activation and emotional processing [50]. Specifically, these processes are likely linked to Alpha/Stability and related traits, traits associated with proneness to aggression and territoriality and control over behavior often in the context of social interactions. Indeed, vasopressin appears to play a role in modulating responses to social cues in a sex-specific manner in a variety of species [25].

### Limitations

There are several limitations to this study. It is important to note that there is a long-standing controversy concerning hierarchical models of personality most recently concerning the question of whether there is a “general factor” of personality (e.g., [7], [51], [52]). While we chose not to address this in the current study, it is important to note that many of the methodological concerns applicable to that debate (e.g., [51], [53]) are potentially relevant to the current study as well. For example, although the Goldberg (2006) method used in the current study has been widely-used in previous studies examining the hierarchical nature of personality, principle components analysis with a varimax rotation may not be the optimal approach for explicating a hierarchical structure. Indeed, in addition to concerns regarding the exploratory nature of such an approach (as opposed to a more confirmatory approach), a hierarchical structure inherently implies correlated factors, an implication that is explicitly disregarded using an orthogonal rotation. As such, although our approach is one of the more widely-used for such situations, it will be important for future research to replicate the current findings with other methodologies.

Limitations notwithstanding, results of the current study confirm the hierarchical structure of personality across species contributing to the larger literature aimed at merging various trait models into a coherent framework. Further, our findings provide evidence for the influence of polymorphisms in the *AVPR1A* gene on not just summed scale scores (e.g., [9]) but on factor-analytically-derived hierarchical personality profiles. Rather than being specific to lower-order, more fine grained personality traits, the influence of the *AVPR1A* gene on personality is at a higher-order two-factor level. Indeed, the genetic effects on the personality traits seems to be consistent across items or traits that load on personality dimensions, independent of the number of personality traits. Along with previous research (e.g., [9], [21], [54], [55]), these findings provide strong support for the existence of biologically-based, evolutionarily derived personality traits among chimpanzees. As we are unable to determine whether the differences that are emerging are due directly to gene expression caused by the presence or absence of the DupB region, it will be important for future research to examine whether variations in the DupB region is associated with differences in the expression of the *AVPR1A* in the brain. In any case, results of the current study provide support for the notion of an evolutionarily-based hierarchical structure of personality and the neurobiological basis of the basic dimensions of personality within this structure in chimpanzees.

## Supporting Information

Checklist S1 **ARRIVE checklist.**
(DOC)
